# Motion corrected water/fat whole‐heart coronary MR angiography with 100% respiratory efficiency

**DOI:** 10.1002/mrm.27732

**Published:** 2019-03-29

**Authors:** Camila Munoz, Gastão Cruz, Radhouene Neji, Rene M. Botnar, Claudia Prieto

**Affiliations:** ^1^ King’s College London School of Biomedical Engineering and Imaging Sciences London United Kingdom; ^2^ Siemens Healthcare, MR Research Collaborations Frimley United Kingdom; ^3^ Pontificia Universidad Católica de Chile Escuela de Ingeniería Santiago Chile

**Keywords:** coronary MRA, respiratory motion correction, water/fat, whole‐heart

## Abstract

**Purpose:**

To develop a framework for respiratory motion‐corrected 3D whole‐heart water/fat coronary MR angiography (CMRA) at 3T with reduced and predictable scan time.

**Methods:**

A 3D dual‐echo acquisition and respiratory motion‐corrected reconstruction framework for water/fat CMRA imaging was developed. The acquisition sequence integrates a 2D dual‐echo image navigator (iNAV), enabling 100% respiratory scan efficiency. Respiratory motion estimated from both the 2D iNAVs and the 3D data itself is used to produce nonrigid motion‐corrected water/fat CMRA images. A first study to investigate which iNAV (water, fat, in‐phase or out‐of‐phase) provides the best translational motion estimation was performed in 10 healthy subjects. Subsequently, nonrigid motion‐corrected water/fat images were compared to a diaphragmatic navigator gated and tracked water/fat CMRA acquisition. Image quality metrics included visible vessel length and vessel sharpness for both the left anterior descending and right coronary arteries.

**Results:**

Average vessel sharpness achieved with water, fat, in‐phase and out‐of‐phase iNAVs was 33.8%, 29.6%, 32.2%, and 38.5%, respectively. Out‐of‐phase iNAVs were therefore used for estimating translational respiratory motion for the remainder of the study. No statistically significant differences in vessel length and sharpness (*P* > 0.01) were observed between the proposed nonrigid motion correction approach and the reference images, although data acquisition was significantly shorter (*P* < 2.6×10^–4^). Motion correction improved vessel sharpness by 60.4% and vessel length by 47.7%, on average, in water CMRA images in comparison with no motion correction.

**Conclusion:**

The feasibility of a novel motion‐corrected water/fat CMRA approach has been demonstrated at 3T, producing images comparable to a reference gated acquisition, but in a shorter and predictable scan time.

## 1 INTRODUCTION

Conventional whole‐heart coronary MR angiography (CMRA) requires the use of fat suppression techniques to produce good‐quality depiction of the vessels, because the coronary arteries are embedded in epicardial fat for most of their course, which can obscure visualization of the vessels if not adequately suppressed. Moreover, high‐intensity signal arising from subcutaneous fat, especially from the chest wall, can result in motion‐induced ghosting, having a detrimental effect in CMRA image quality.

The most common approach for fat suppression in CMRA is based on the use of a spectral presaturation with inversion recovery (SPIR) pulse before data acquisition to null or minimize the fat signal, which relies on the precession frequency and relaxation time differences between water and fat. Although this technique has been shown to be reliable and robust at 1.5T, it can produce suboptimal results at higher magnetic field strengths, because of its intrinsic sensitivity to inhomogeneities in both the static and the B_1_ transmit magnetic fields. Alternatively, the difference in precession frequency between water and fat can be exploited by acquiring images at multiple echo times and then combining them to produce images where the two species are fully separated, using so‐called water/fat separation techniques, as originally proposed by Dixon.^1^

Dixon‐based CMRA imaging has been shown to improve image quality at 1.5T^2^ and 3T^3^ compared to conventional SPIR fat suppression, increasing signal‐to‐noise ratio (SNR) of the blood and improving blood‐to‐fat contrast‐to‐noise ratio in the water images. The water/fat separation approach for CMRA has also the benefit of providing a complementary fat image that carries additional diagnostic information: Studies have suggested that increased epi‐ and pericardial fat volumes are associated with increased cardiovascular risk, including increased vascular calcification, luminal stenosis and plaque burden, and increased likelihood of adverse cardiovascular events.^4^, ^5^, ^6^, ^7^ Furthermore, water/fat cardiac MR imaging has shown promising results for the assessment of fibro‐fatty infiltration in the myocardium and cardiac masses,^8^, ^9^ and a modified Dixon approach has been recently demonstrated to enable 3D late gadolinium enhancement imaging in a single breath‐hold^10^ at 1.5T. These developments suggest a growing interest in the use of Dixon‐based techniques for cardiac imaging, because of both the improvement in image quality arising from the enhanced fat suppression and gain in SNR and the potential additional clinical information offered by the fat image.

The water/fat CMRA imaging approaches that have been proposed in the literature make use of 1D diaphragmatic navigators for respiratory gating and tracking, to allow for free‐breathing volumetric whole‐heart acquisitions.^2^, ^3^ Although 1D navigators produce good‐quality images, they are based on a simplified linear respiratory motion model, typically using a constant scaling factor of 0.6 to compensate only for superior‐inferior translational motion. This scaling factor is, however, subject specific, and the diaphragmatic‐to–heart motion relationship can be nonlinear because of hysteresis effects. In addition, the gating efficiency depends on the breathing pattern of the subject being scanned. For subjects with irregular breathing patterns, the gating efficiency tends to be low, leading to prolonged and unpredictable acquisition times thereby limiting the clinical translation of the technique. In order to facilitate the integration of 3D Dixon‐based approaches into the cardiac MR clinical routine, more advanced respiratory motion compensation techniques are required.

We have recently proposed a combined beat‐to‐beat translational and bin‐to‐bin nonrigid respiratory motion correction approach for fat‐suppressed 3D whole‐heart single‐echo CMRA^11^, ^12^, ^13^ and other whole‐heart applications.^14^ Here, we extend this approach to dual‐echo water/fat CMRA acquisition and reconstruction scheme with 100% respiratory scan efficiency. Dual‐echo low‐resolution 2D image navigators (iNAVs) provide water, fat, in‐phase and out‐of‐phase images from which 2D beat‐to‐beat translational respiratory motion can be estimated. Nonrigid respiratory motion is estimated from high‐resolution 3D out‐of‐phase CMRA images reconstructed at different respiratory positions (so‐called respiratory bins). Motion‐corrected in‐ and out‐of‐phase high‐resolution 3D images are then produced by combining a beat‐to‐beat 2D translational motion correction in the foot‐head (FH) and right‐left (RL) directions that captures the temporal resolution of the respiratory‐induced motion of the heart, and a bin‐to‐bin 3D nonrigid motion correction that captures the complex spatial deformation of the heart during the entire respiratory cycle. This approach uses all the acquired data for image reconstruction, and it has a predictable scan time.

A first study to investigate the accuracy of the 2D translational motion estimated from the different iNAVs obtained with the dual‐echo acquisition (i.e., fat, water, in‐phase and out‐of‐phase navigators) was performed in 10 healthy subjects at 3T. After identifying the iNAV that produced the best translational motion estimates, the nonrigid motion‐corrected water/fat CMRA reconstruction was tested and compared to a water/fat reference scan with diaphragmatic navigator gating and tracking with matching imaging parameters.

## 2 METHODS

### 2.1 Image acquisition

**Figure 1:**
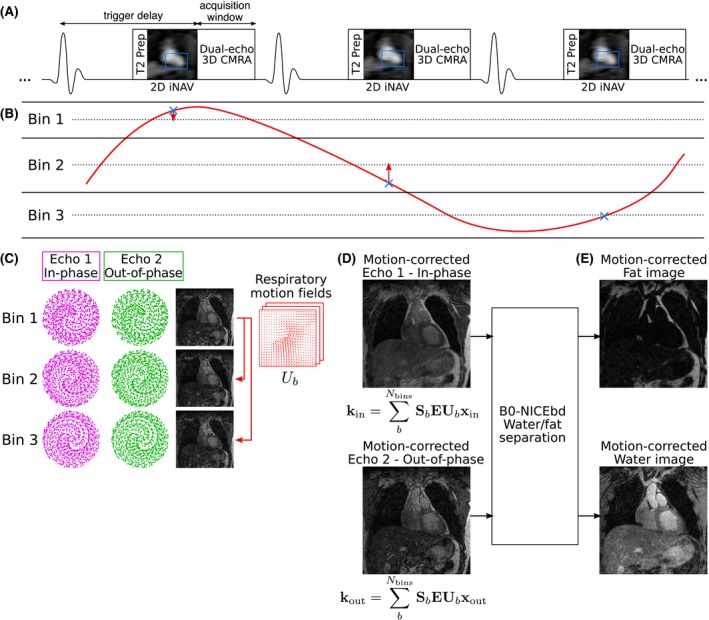
Water/fat CMRA acquisition and reconstruction scheme. (A) Dual‐echo CMRA data is acquired following a golden‐step spiral ordering in a Cartesian grid. A low‐resolution coronal 2D dual‐echo iNAV is acquired before CMRA acquisition, for respiratory motion estimation; and a T_2_ preparation (T2 Prep) pulse is used to enhance the contrast between the coronary arteries and myocardium. (B) FH motion estimated from the 2D iNAVs is used to define a number of respiratory bins and correct the data to the center of the corresponding bin (red arrows). (C) Dual‐echo CMRA data are binned, and echo images reconstructed at different respiratory positions are used to estimate nonrigid deformation fields. (D) This nonrigid motion is used to reconstruct motion‐corrected echoes, which are finally used as an input into (F) the B0‐NICEbd water/fat separation algorithm to obtain motion‐corrected water and fat CMRA images

The proposed acquisition sequence consists of an ECG triggered free‐breathing dual‐echo 3D gradient echo CMRA sequence as shown in Figure 1A (a detailed pulse sequence diagram can be found in Supporting Information Figure S1). The dual‐echo CMRA data are acquired following a fully sampled golden‐step Cartesian spiral profile order (CASPR) sampling trajectory,^15^ so that one spiral interleaf is acquired at each heartbeat for both in‐ and out‐of‐phase echo times. A low‐resolution coronal 2D dual‐echo iNAV is acquired by adding spatially encoded low flip‐angle lines before the 3D dual‐echo CMRA acquisition.

### 2.2 Image reconstruction

The image reconstruction scheme extends the method proposed in previous works^11^, ^12^ to a dual‐echo acquisition as follows. In the first step, the FH motion estimated from the 2D iNAVs (water, fat, in‐phase or out‐of‐phase) is used to define a number of respiratory bins, each containing approximately the same amount of data, and the 3D dual‐echo k‐space data acquired at each heart beat are corrected to the centre of the corresponding bin by applying a linear phase in the FH and RL directions (Figure 1B). In the second step, 3D dual‐echo images are reconstructed for each respiratory bin using a soft‐gated iterative sensitivity encoding^16^, ^17^ approach (Figure 1C). The 3D out‐of‐phase bin images are used to estimate 3D nonrigid deformation fields by free‐form image registration,^18^ using the end‐expiratory bin as the reference position. In the third step, motion‐corrected images are reconstructed separately for each echo incorporating the nonrigid deformation fields in a generalized matrix description (GMD) approach,^19^ by solving Equation 1 for **x** *_e_* (Figure 1D):

(1) $$k_{e}=\sum_{b}S_{b}{\mathrm{EU}}_{b}x_{e}$$

where **x** *_e_* are the images for each echo (*e* = in‐phase, out‐of‐phase), **k** *_e_* is the translationally corrected k‐space for echo *e*, **E** is the forward encoding operator, including the discrete Fourier transform and coil sensitivities, **U** *_b_* is the nonrigid motion operator that transforms an image from the reference position to any position *b*, and **S** *_b_* corresponds to the sampling matrix containing the k‐space points acquired at respiratory bin *b*.

In the fourth and final step of the reconstruction (Figure 1E), water and fat images are obtained with the B0‐NICEbd method proposed by Liu et al^20^ for water/fat separation of dual‐echo acquisitions performed with bipolar gradients. This method assumes that the total difference in phase between the in‐ and out‐of‐phase images, **φ** *_io_*, arises from three sources: first, the gain in phase between echoes due to the water/fat chemical shift; second, a phase due to field inhomogeneities, **φ** *_B_*_0_; and third, a linear phase in the frequency encoding direction due to the bipolar readout gradient, **φ** *_bi_*. The B0‐NICEbd method estimates each of these phase components separately and then uses Equation 2 to obtain the water and fat images, **x** *_w_* and **x** *_f_*, respectively, with $\Delta \varphi =\varphi_{\mathrm{io}}-\varphi_{B0}-\varphi_{\mathrm{bi}}$ representing the phase induced only by the chemical shift between fat and water, and **x** *_in_* and **x** *_out_* indicating the in‐ and out‐of‐phase images, respectively.

(2) $$\begin{array}{c} x_{w}=\frac{\left|\left|x_{\mathrm{in}}\right|+\left|x_{\mathrm{out}}\right|e^{-i\Delta \varphi }\right|}{2}\\ x_{f}=\frac{\left|\left|x_{\mathrm{in}}\right|-\left|x_{\mathrm{out}}\right|e^{-i\Delta \varphi }\right|}{2} \end{array}$$

### 2.3 Experiments

Ten healthy subjects (age 30.2 ± 2.8 years; 4 male) were scanned under free breathing using a prototype implementation of the proposed dual‐echo 3D CMRA sequence, with the following imaging parameters: coronal orientation, RL phase encoding, bipolar readout acquisition, resolution = 1.3 mm isotropic, field of view (FOV) = 312 × 312 × 78 to 104 mm covering the whole heart, flip angle = 20°, TR/TE1/TE2 = 5.26/2.46/3.69 ms, bandwidth = 1302 Hz/pixel, and T_2_ preparation with a duration of 50 ms. The 2D dual‐echo iNAVs were acquired with the following parameters: high‐low Cartesian trajectory, coronal orientation, RL phase encoding, 14 low flip‐angle lines (flip angle = 3º), same TR/TE1/TE2 and FOV as the 3D CMRA acquisition, resulting in an acquired resolution of 1.3 × 22.8 mm, interpolated to 1.3 × 1.3 mm during reconstruction. A subject‐specific trigger delay and acquisition window (95–115 ms, corresponding to 18–22 lines in k‐space) was set coinciding with the mid‐diastolic rest period.

An additional Cartesian ECG‐gated dual‐echo 3D CMRA scan with a 1D diaphragmatic respiratory gating and tracking, with a 6‐mm gating window, tracking factor of 0.6,^21^ and adaptive acceptance window position, with matching imaging parameters was performed for comparison purposes. Because of time constraints, this acquisition was performed using 2 ×‐accelerated GRAPPA^22^ to reduce scan time, using 24 calibration lines.

All acquisitions were performed on a 3T Biograph mMR scanner (Siemens Healthcare, Erlangen, Germany). Written informed consent was obtained from all subjects according to institutional guidelines, and the institutional ethics committee approved the study.

### 2.4 Performance of image navigators

Because the 2D iNAVs are acquired with a dual‐echo readout, image navigators with different contrasts can be reconstructed. The performance of water, fat, in‐phase and out‐of‐phase iNAVs for 2D translational motion estimation and correction was compared. For this purpose, FH and RL motion were estimated from each iNAV using the same tracking template around the heart. Then, 3D CMRA in‐ and out‐of‐phase images were reconstructed with 2D translational motion correction only and were used to obtain translationally corrected water/fat CMRA images using the B0‐NICEbd method.

Reconstructed images were reformatted along the right (RCA) and left anterior descending (LAD) coronary arteries and assessed in terms of presence of extreme blurring in either coronary and/or presence of artefacts attributed to residual water/fat swaps. The reconstructed images were also visually compared to the 1D gated and tracked water/fat reference images. Furthermore, vessel sharpness of both RCA and LAD coronary arteries was computed in all translational motion corrected images, using dedicated software.^23^ Vessel sharpness values were normalized to the signal intensity of the center line of each vessel, so that 100% sharpness refers to a maximum signal intensity change at the vessel edge.

### 2.5 Motion‐corrected water/fat CMRA

After finding the best iNAV for FH and RL translational estimation, three reconstructions were performed for each acquired data set: (1) the proposed translational plus nonrigid respiratory motion‐corrected reconstruction (TC+GMD); (2) 2D translational motion correction only (TC); and (3) without motion correction (NMC) for comparison purposes. The number of respiratory bins for TC+GMD is defined automatically such that there are between 3 and 5 bins; each bin contains approximately the same amount of data and has a maximum bin width of 4.5 mm.^11^, ^12^ All reconstructions, including water/fat separation, were performed offline in MATLAB (The Mathworks, Inc., Natick, MA). The water/fat CMRA data acquired with 1D diaphragmatic respiratory gating and tracking (called Gated hereafter) were reconstructed with the scanner software, including water/fat separation, for comparison purposes.

### 2.6 Image analysis

Water CMRA images were reformatted to simultaneously visualise the RCA and LAD artery, using dedicated software,^23^ and the same reformatting was applied to the corresponding fat images. Image‐quality metrics of visible vessel length and sharpness were obtained for the water images only, with sharpness computed for both the first 4 cm and the whole visible length of each vessel. The difference in resulting image quality metrics (vessel length and sharpness) between each reconstructed image and the reference Gated CMRA was evaluated with a paired *t *test, with a *P* value of 0.01 considered statistically significant.

## 3 RESULTS

Scans were successfully completed in all subjects. The average acquisition time for the proposed fully sampled motion‐corrected water/fat CMRA sequence was 15.29 ± 1.13 minutes, while the average acquisition time for the 2×‐accelerated water/fat Gated CMRA acquisition was 26.05 ± 5.51 minutes. For the proposed motion‐corrected approach, 3.8 ± 0.8 respiratory bins were used on average. The minimum, maximum, and average respiratory efficiency of the Gated CMRA acquisition was 32%, 63%, and 47.3±11.5%, respectively, for a 6‐mm gating window.

### 3.1 Performance of image navigators

**Figure 2:**
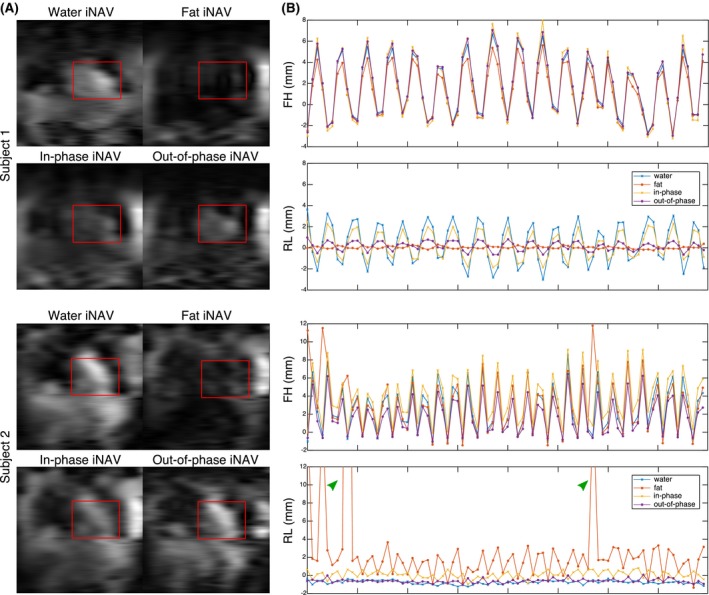
(A) Example iNAVs for 2 subjects, showing water, fat, in‐phase and out‐of‐phase navigators, with the corresponding template used for motion tracking indicated in red. In both cases, water/fat separation results in a clear depiction of subcutaneous fat in the fat iNAVs. Reduced spatial resolution prevents the visualization of smaller fatty structures. (B) FH and RL motion estimation from water, fat, in‐phase and out‐of‐phase iNAVs for the same subjects. In both cases, FH estimation is consistent across different iNAVs, while differences are apparent in RL estimation. In particular for subject 2, fat iNAVs result in unrealistic RL motion estimation (>12 mm, green arrows)

Figure 2A shows water, fat, in‐phase and out‐of‐phase iNAVs for two representative healthy subjects. It can be observed that subcutaneous fat is adequately separated in the water/fat iNAVs in both cases, while smaller fat structures, such as epi‐ and pericardial fat, are not clearly depicted in the fat iNAVs.

The presence and distribution of cardiac fat substantially affects the estimation of translational motion, as can be observed in Figure 2B, that shows the FH and RL motion estimated from each of the reconstructed iNAVs. It can be observed that while the estimation of FH motion is similar across the different navigators in both cases, differences are apparent in the RL motion estimation. In subject 1, water and in‐phase iNAVs result in larger RL estimates compared to fat and out‐of‐phase iNAVs. A more extreme case can be observed for subject 2, where the lack of defined structures in the fat iNAVs resulted in unrealistic RL motion estimation, also impacting the accuracy of FH motion estimation.

**Figure 3:**
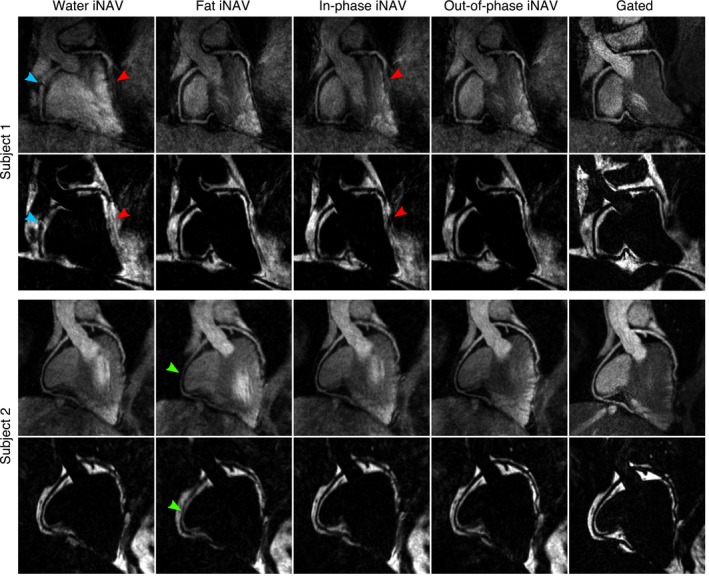
Reformatted translationally motion‐corrected water and fat images for 2 subjects, with motion estimated from water, fat, in‐phase and out‐of‐phase iNAVs, alongside corresponding Gated images. Water and in‐phase iNAVs resulted in blurring and artefacts for subject 1 (red and blue arrows), while fat iNAVs resulted in blurring in subject 2 (green arrows). Out‐of‐phase iNAVs resulted in good image quality for both subjects, with a delineation of the coronary arteries and depiction of the fat tissue comparable to the corresponding water and fat Gated images

Figure 3 shows reformatted water and fat images obtained after translational motion correction for the same two subjects, with FH and RL motion estimated from each of the iNAVs, alongside the corresponding Gated images. From both the water and fat reformatted images, it can be observed that for subject 1, water and in‐phase iNAVs result in blurring in the mid and distal segments of the LAD (red arrows), preventing the visualization of the vessel, while additional artefacts can be observed in the RCA when using water iNAVs (light blue arrow). On the contrary, when using fat or out‐of‐phase iNAVs, the resulting image quality improves, with no apparent artefacts and a depiction of the coronary arteries visually similar to the one observed in the Gated images. On the contrary, for subject 2, water and in‐phase iNAVs result in similar image quality, with no presence of localized artefacts and sufficient sharpness to visualize the vessels in the resulting water images. When using fat iNAVs, however, the RCA appears blurred (green arrows), because of errors in motion estimation. Finally, for both subjects, using out‐of‐phase iNAVs results in a slight improvement in vessel sharpness, particularly in the LAD, compared to water or in‐phase iNAVs.

When considering the presence of extreme blurring and/or local water/fat swaps in the reformatted images, we observed that translational motion‐corrected images based on water, fat, and in‐phase iNAVs presented artefacts in 3, 9, and 5 of the 20 vessels studied, respectively. Conversely, translational motion‐corrected images based on out‐of‐phase iNAVs were free from artefacts in all cases. Average vessel sharpness for both coronaries in the water CMRA images when using water, fat, in‐phase and out‐of‐phase iNAVs was 33.8 ± 6.9%, 29.6 ± 8.5%, 32.2 ± 7.2%, and 38.5 ± 5.9%, respectively.

Overall, out‐of‐phase iNAVs produced sharper water/fat translational motion corrected CMRA images without visible artefacts. Out‐of‐phase iNAVs were therefore used for estimating beat‐to‐beat translational motion for the remainder of this study.

### 3.2 Motion‐corrected water/fat CMRA

**Figure 4:**
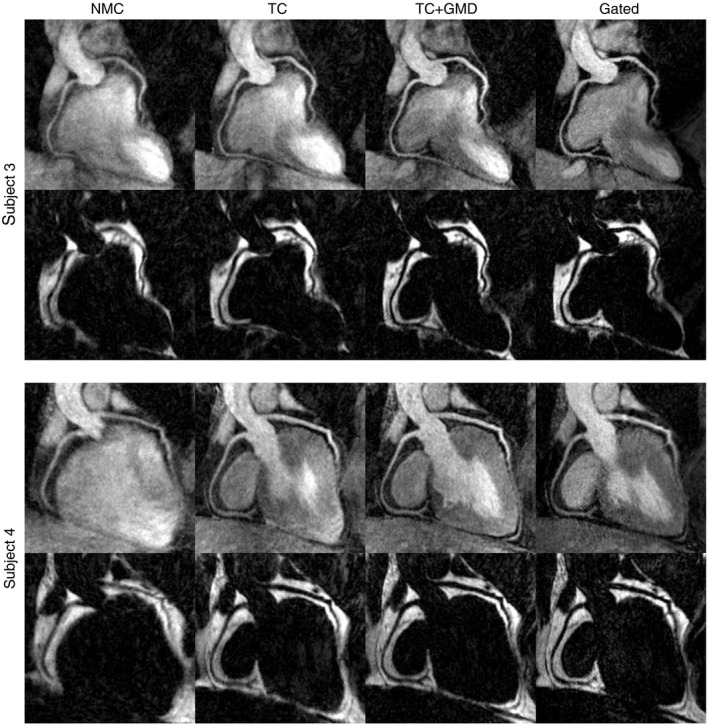
Reformatted CMRA water/fat images for 2 representative subjects showing uncorrected (NMC), translational motion‐corrected (TC), translational plus nonrigid motion‐corrected (TC+GMD), and Gated images. TC improves depiction of both the RCA and LAD in all cases. Further improvements are obtained with the TC+GMD approach, which results in images comparable to the Gated images

Figure 4 shows reformatted water/fat CMRA images for two representative subjects for the NMC, TC, and TC+GMD reconstructions, alongside Gated images for comparison purposes (an additional case can be observed in Supporting Information Figure S2). For all subjects, an improved delineation of the RCA and LAD can be observed in the water CMRA images after applying TC, in terms of visible length and depiction of the vessels. Further improvements are observed when using TC+GMD, particularly in the depiction of the distal segments of the arteries, resulting in images that are visually comparable to the Gated images. Similarly, in the corresponding cardiac fat images, it can be observed that TC and TC+GMD improve depiction of the epicardial fat compared to NMC images, and delineation of the fat surrounding the vessels is also significantly improved. A video showing the whole‐heart motion‐corrected water/fat CMRA images for a representative subject is provided in Supporting Information Video S1.

**Figure 5:**
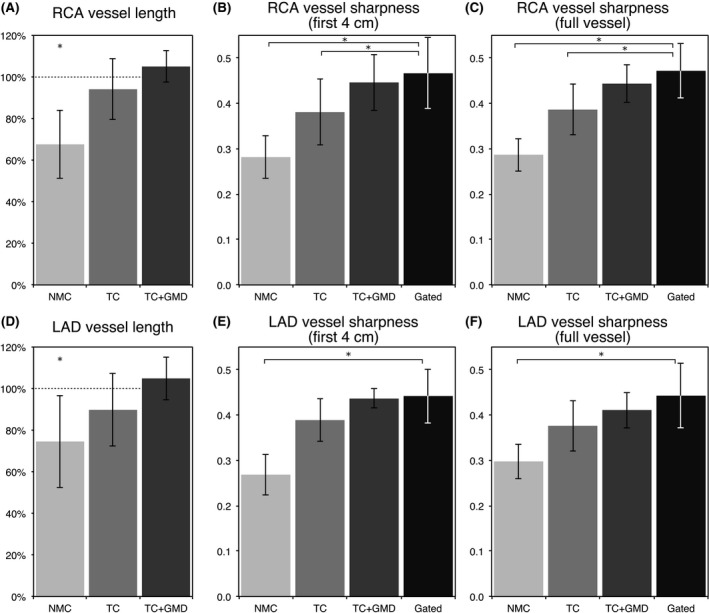
Image‐quality metrics for the RCA and LAD arteries for 10 healthy subjects, for NMC, TC, TC+GMD, and Gated water images. The metrics included: visible vessel length along the (A) RCA and (D) LAD, where each measure is normalized to the length observed in the corresponding Gated image; vessel sharpness for the (B,C) RCA and (E,F) LAD, for both the first 4 cm and full length of each vessel. ^*^Denotes a statistically significant difference with *P *< 0.01 compared to the Gated images

Image quality metrics obtained from the water CMRA images are summarized in Figure 5 and Supporting Information Table S1, which shows that no statistically significant differences were observed between the Gated and the TC+GMD approach for any of the obtained metrics. The observed vessel length for TC+GMD images was slightly higher than the one obtained from Gated images. In particular, visible length of RCA and LAD in the TC+GMD images was, on average, 105.0 ± 7.5% and 104.9 ± 10.3% of the length measured in Gated images, respectively. Lower values were obtained for TC and NMC, with an average of 94.1 ± 14.5% and 67.6 ± 16.6% for the RCA and 89.8 ± 17.3% and 74.6 ± 22.1% for the LAD, respectively. Statistically significant differences were found in the visible vessel length between NMC and Gated images for both coronaries.

A similar trend was observed when analyzing the sharpness of the coronaries. The average vessel sharpness for the proximal segment (first 4 cm) and the full length of the vessels obtained from the TC+GMD reconstructions was slightly lower than the one obtained from Gated images; however, such difference was not found to be statistically significant (Supporting Information Table S1). On the contrary, statistically significant differences between Gated and NMC were obtained when analyzing either the proximal segment or the full length of both the RCA and LAD. Furthermore, significant differences were also observed in vessel sharpness between Gated and TC for the RCA only.

## 4 DISCUSSION

In this study, we present a framework for respiratory motion‐corrected water/fat CMRA with 100% scan efficiency, which allows shorter and predictable scan time. This approach extends the respiratory motion‐corrected acquisition and reconstruction presented in previous works^11^, ^12^ to a dual‐echo sequence, performing translational plus nonrigid motion correction in order to obtain respiratory motion‐corrected echo images. Then, these echo images are used to obtain water and fat cardiac CMRA images by using a water/fat separation algorithm designed specifically for dual‐echo acquisitions with bipolar readout gradients.^20^

In a first study, the performance of the different iNAVs that can be obtained from dual‐echo acquisitions for estimating 2D translational motion was investigated. Results showed that subcutaneous fat is adequately separated in the water/fat iNAVs; however, because the iNAVs are a projection in the anterior‐posterior direction and have a low spatial resolution, smaller fat structures are not clearly depicted. After performing motion tracking, it was observed that FH motion estimation was similar for the different image navigators, whereas apparent differences were observed in RL motion estimation, favoring the out‐of‐phase iNAVs.

The iNAVs are designed to provide more accurate motion information in the main direction of the respiratory‐induced motion of the heart. Therefore, they are acquired with high‐resolution in the FH direction, resulting in stable and consistent motion estimation across navigators. To limit the duration of the iNAVs to approximately 50 ms, iNAVs are acquired with only 14 phase encoding steps and thus have low spatial resolution in the RL direction, so that changes in tissue contrast in this direction can have a significant impact on motion estimation.

For subjects with a larger amount of adipose tissue around the heart, such as subject 1 in Figure 2, fat iNAVs were shown to provide accurate RL motion, outperforming water and in‐phase iNAVs, which resulted in blurring and artefacts attributed to erroneous motion estimation. However, for subjects with less visceral and/or cardiac fat, such as subject 2 in Figure 2, fat iNAVs resulted in erroneous RL motion estimation, impacting the quality of translationally motion‐corrected images. Furthermore, water and in‐phase iNAVs were shown to perform better in the latter cases. In contrast, out‐of‐phase iNAVs were observed to perform consistently better in all studied cases. This is likely due to the high‐contrast black rim artefact between myocardium and epicardial fat produced by the chemical shift between water and fat, which is tracked by the image registration algorithm.

Performance of the translational correction only (TC) and the translational plus nonrigid motion correction (TC+GMD), based on out‐of‐phase iNAVs, was compared to uncorrected (NMC) images and to a reference diaphragmatic 6‐mm gated and tracked acquisition. In all cases, improvements are apparent in the delineation of the coronary arteries when using TC compared to NMC, and additional improvements are observed when using the TC+GMD approach. Metrics of image quality showed no statistically significant differences between TC+GMD and Gated water CMRA images for the visible vessel length and vessel sharpness for both the right and left coronary arteries, while the scan time was predictable and significantly shorter (*P* < 2.6 × 10^–4^). It is worth noting that the fully sampled CASPR trajectory results in an elliptical sampling mask in the phase encoding plane,^15^ which reduces scan time compared to traditional fully sampled Cartesian acquisitions by approximately 20%.

These results are consistent with previous findings in conventional fat‐suppressed CMRA imaging using the TC+GMD approach at 1.5T^11^ and 3T.^12^ This indicates that the 2D iNAV‐based TC+GMD approach is robust and reliable for coronary artery imaging, and can be used with different image contrasts and in different sequence configurations.

This study has some limitations. First, the proposed approach used conventional in‐ and out‐of‐phase echo times to produce the water/fat CMRA images. This restriction in the echo times resulted in an increased TR compared to conventional fat‐suppressed CMRA approaches. In future studies, this problem can be alleviated by using flexible TE approaches^24^, ^25^ that allow the echo images not to be acquired at exact in‐ and out‐phase times, enabling the use of reduced TEs and therefore, resulting in reduced TRs. Alternatively, shorter and/or asymmetric excitation pulses can be used to enable the use of the shortest out‐of‐phase echo time (around TE = 1.23 ms), thereby reducing the TR to ~4 ms and enabling shorter acquisitions. Second, although the image resolution used in this study was sufficient to depict the coronary arteries in healthy subjects, in clinical patients a higher spatial resolution may be required for accurate detection of vessel abnormalities, including stenosis or small aneurysms. Future work includes acceleration of the introduced approach using variable density trajectories that allow undersampled acquisitions, such as the ones described in previous works,^26^, ^27^ in order to increase spatial resolution and anterior‐posterior coverage without increasing acquisition time. Furthermore, in this study, diaphragmatic gated and tracked images were used for comparison purposes, because this remains the reference technique for respiratory motion compensation in cardiac MR in clinical practice. However, unpredictable and prolonged scan times attributed to low respiratory gating efficiency may result in image degradation. Further studies including comparison between the motion‐corrected water/fat CMRA images and a gold‐standard imaging technique, such as computed tomography coronary angiography or invasive x‐ray angiography, are required to validate the technique in patients with cardiovascular disease.

## 5 CONCLUSION

A novel framework for respiratory motion‐corrected water/fat coronary MR angiography at 3T has been presented, enabling 100% respiratory scan efficiency and predictable scan time. No statistically significant differences were found between the proposed approach and a reference diaphragmatic gated and tracked acquisition when quantifying image quality in the water CMRA images, in terms of visible length and sharpness of the vessels, while scan time for the proposed approach was significantly shorter. Future work includes accelerating the acquisition for improved spatial resolution and coverage, and validating the framework in patients with cardiovascular disease.

## Supporting information


**VIDEO S1** Motion corrected water and fat CMRA images for a representative subject

**FIGURE S1 **Pulse sequence diagram, showing RF excitations and readout gradient, indicating echo times TE1/TE2. The intervals where data are acquired are indicated in red
**FIGURE S2 **Reformatted CMRA water/fat images for an additional representative subject showing uncorrected (NMC), translational motion‐corrected (TC), translational plus nonrigid motion‐corrected (TC+GMD), and Gated images
**TABLE S1 **Image‐quality metrics for the RCA and LAD arteries for NMC, TC, TC+GMD, and Gated water images, including vessel length and vessel sharpness, for both the first 4 cm and full length of each vessel *P* values compared to the Gated images are indicated for each case. RCA = right coronary artery; LAD = left anterior descending artery; NMC = no motion corrected; TC = 2D translational motion corrected; TC+GMD = 2D translational plus 3D nonrigid motion corrected; Gated = 1D diaphragmatic navigator gated and tracked acquisition
