# Supplementary material for: Motion corrected water/fat whole‐heart coronary MR angiography with 100% respiratory efficiency
Source: Magn Reson Med. 2019 Mar 29;82(2):732–42. doi: 10.1002/mrm.27732 (PMC6563440; doi:10.1002/mrm.27732)
Supplement: Supplementary file 2 — FIGURE S1 Pulse sequence diagram, showing RF excitations and readout gradient, indicating echo times TE1/TE2. The intervals where data are acquired are indicated in red FIGURE S2 Reformatted CMRA water/fat images for an additional representative subject showing uncorrected (NMC), translational motion‐corrected (TC), translational plus nonrigid motion‐corrected (TC+GMD), and Gated images TABLE S1 Image‐quality metrics for the RCA and LAD arteries for NMC, TC, TC+GMD, and Gated water images, including vessel length and vessel sharpness, for both the first 4 cm and full length of each vessel P values compared to the Gated images are indicated for each case. RCA = right coronary artery; LAD = left anterior descending artery; NMC = no motion corrected; TC = 2D translational motion corrected; TC+GMD = 2D translational plus 3D nonrigid motion corrected; Gated = 1D diaphragmatic navigator gated and tracked acquisition [file MRM-82-732-s002.pdf]

Image Metrics		NMC	TC	TC+GMD	Gated
Normalised vessel length					
RCA	mean±SD	67.6±16.6%	94.1±14.5%	105.0±7.5%	NA
	p-value	3.18×10^{-4}*	1.87×10^{-1}	7.91×10^{-2}	
LAD	mean±SD	74.6±22.1%	89.8±17.3%	104.9±10.3%	NA
	p-value	5.29×10^{-3}*	9.93×10^{-2}	1.73×10^{-1}	
Vessel sharpness (first 4 cm), a.u.					
RCA	mean±SD	0.282±0.047	0.381±0.072	0.446±0.062	0.467±0.078
	p-value	1.83×10^{-5}*	2.75×10^{-4}*	1.60×10^{-1}	
LAD	mean±SD	0.269±0.045	0.389±0.047	0.436±0.021	0.442±0.059
	p-value	2.70×10^{-5}*	4.14×10^{-2}	7.60×10^{-1}	
Vessel sharpness, a.u.					
RCA	mean±SD	0.287±0.036	0.386±0.056	0.444±0.041	0.472±0.060
	p-value	1.25×10^{-5}*	7.08×10^{-4}*	5.63×10^{-2}	
LAD	mean±SD	0.298±0.038	0.376±0.056	0.411±0.039	0.443±0.071
	p-value	2.28×10^{-4}*	1.80×10^{-2}	1.50×10^{-1}	

*p-value < 0.01 vs. Gated

Supporting Information Table S1. Image quality metrics for the RCA and LAD arteries for NMC, TC, TC+GMD and Gated water images, including vessel length and vessel sharpness, for both the first 4 cm and full length of each vessel. p-values compared to the Gated images are indicated for each case. RCA: right coronary artery, LAD: left anterior descending artery. NMC: no motion corrected, TC: 2D translational motion corrected, TC+GMD: 2D translational plus 3D non-rigid motion-corrected, Gated: 1D diaphragmatic navigator gated and tracked acquisition.

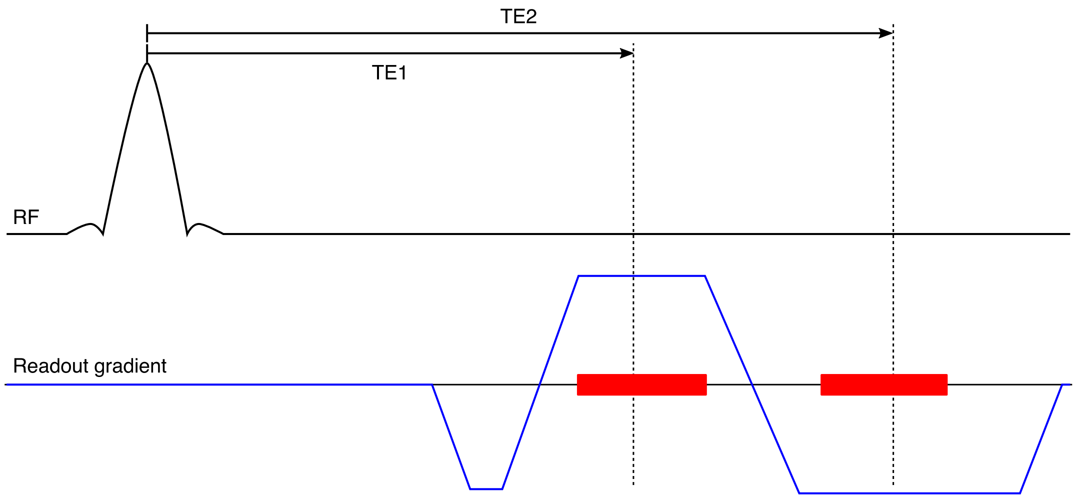

Supporting Information Figure S1. Pulse sequence diagram, showing RF excitations and readout gradient, indicating echo times TE1/TE2. The intervals where data is acquired are indicated in red.

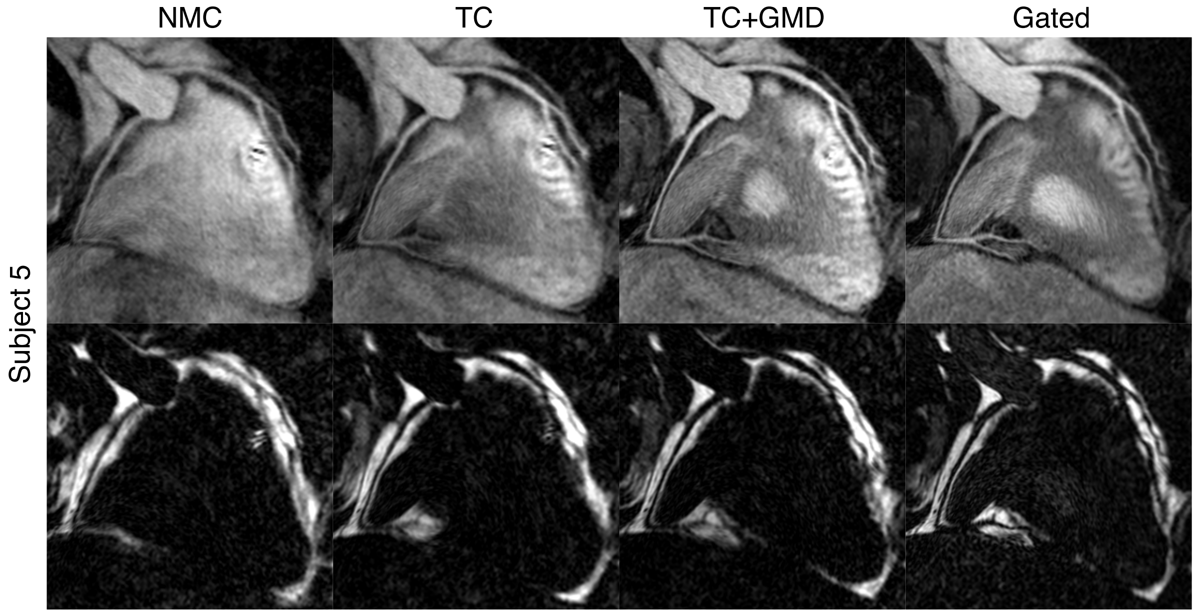

Supporting Information Figure S2. Reformatted CMRA water/fat images for an additional representative subject showing uncorrected (NMC), translational motion-corrected (TC), translational plus non-rigid motion-corrected (TC+GMD) and Gated images
